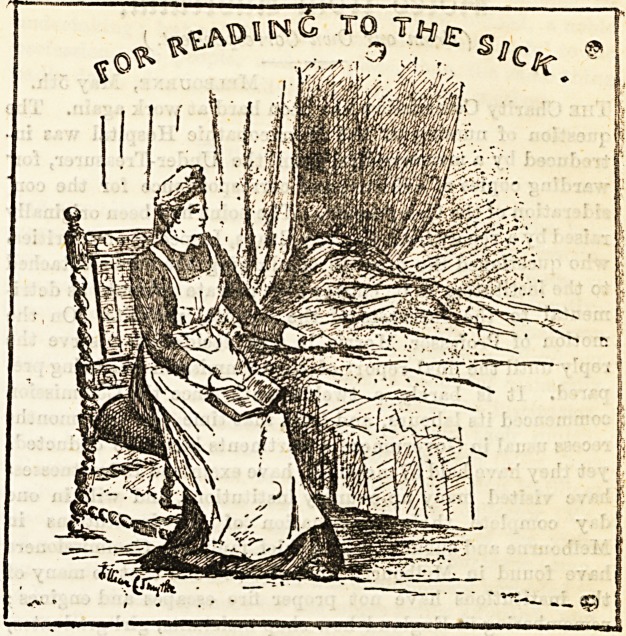# The Hospital Nursing Supplement

**Published:** 1891-06-27

**Authors:** 


"The Hospital" Nursing Mirror.

Being the Extra Nursing Supplement of "The Hospital" Newspaper.

Contributions for this Supplement should be addressed to the Editor, The Hospital, 140, Strand, London, W.C., and should have the word "Nursing" plainly written in left-hand top corner of the envelope.

### En Passant.

Sick-Nursing at Stalybridge.—It is just ten years since Mrs. Knott promoted a sick nursing and mothers' help scheme for Stalybridge, and now there are two nurses and a midwife kept constantly at work, and the whole expenses for the last year were only £166. We are glad to observe that Nurse Twamley continues her work for the Society; we do not quite understand the position of Nurse Jones, who is apparently untrained. One valuable adjunct to the society is the numerous appliances, such as feeding cups, bath chairs, &c., which are lent to patients in return for a small weekly payment.

Benefit Association.—This society for providing nurses for the sick at Reigate, Redhill, and some adjoining parishes, issues a good report of last year's work. The staff has been increased to from twelve to sixteen nurses, and 200 patients have been nursed. The scheme of this association is excellent; subscribers pay an annual subscription of from 2s. a year for labourers to 21s. a year for "affluent" persons. Then subscrsbers can have the nurses' services for a fee of from 1s. to 15s. a week. The nurse does not go more than two miles from her home, and she attends confinements. In this way good nursing is secured to the middle class, who, not choosing to accept the charity of a district nurse, yet not able to pay the high fees demanded by private institutions, are most often left to lay and voluntary nurses.

Richmond House.—Such is the name Mrs. St. Horton has given to the Convalescent Home and Nursing Institution which she has just opened at Worthing. The house is pleasantly situated in a healthy position, and has a large garden and tennis ground. The institution has been opened for the reception of patients requiring medical treatment and convalescents who can afford to make small weekly payments. Infectious cases are not received. The Institution is entirely carried on by voluntary workers, at the head of whom is the honorary lady superintendent, Mrs. St. A. Horton, whose experience renders her thoroughly qualified for the discharge of the duty she has so kindly undertaken. Mr. W. J. Harris has identified himself with the institution as the Honorary Medical Officer. The opening ceremony was one of prayer and song, and in the evening an entertainment was given to the patients.

Northampton Nurses.—The annual meeting of the Northamptonshire Nursing Institution was held under the presidency of the Bishop of Leicester. The report stated that since the last meeting the house known as 31, Hazelwood-road had been added to Nos. 33 and 35, which already formed the headquarters of the institution, and that the tenancy of No. 54 had been relinquished. The three houses, which had practically been changed into one large residence, were taken at an annual rent of £75. Henceforward they anticipated the pleasure of maintaining a larger staff of nurses, and in view of this several probationers had been through a course of training in the Northampton Infirmary, and also an additional training a very practical character in the district nursing of the town. The present staff of nurses numbered 18, and the district visits—7,313 in number—were in excess of those of the previous year. The private nurses, when at home, had always been ready to help and to sit up at night in urgent or dying cases, and over and over again the friends of the patients had called or written to express their gratitude for the services of the nurses. Whole families with influenza had been attended during the epidemic, besides which nurses had been supplied for a fresh outbreak of diphtheria at East Haddon, for typhus and enteric fever at Desborough, and for scarlet and typhoid fever at Kettering. The report having been adopted, the balance-sheet was read by Mrs. Burnham. From this it appear that the year was commenced with a total balance in hand of £226 9s. 11½d. Subscriptions during the year amounted to £97 14s. 6d., donations to £73 16s., nurses' earnings to £571 4s. 6d., payments from patients in the house to £24 11s. 6d., altogether £1,021 18s. 5½d. On the other hand, the expenditure was such as to leave a balance of £78 8s. 1d. In the course of a short discussion upon the balance-sheet, Miss Stewart suggested that the probationers should be trained at the infirmary for nothing. At present it cost the nursing institution £20 a-year for each nurse trained there. The probationers, she pointed out, gave the infirmary the advantage of their services, and when they had done training it was very often the townspeople they nursed who subscribed so much to the infirmary. They did not subscribe very much to the nursing institution.

The New Departure at Queen's.—Considerable feeling is being displayed in Birmingham over the proposal to add a private nursing department to the Queen's Hospital; but the scheme is going to be tried for three years, and friends have come forward and guaranteed the hospital against loss for that period. The objectors say that the scheme will injure the favourite institution which has worked so long and so well at the Crescent; they also say it will be a case of "sweating" the nurses. The views of the promoters of the scheme are well given by Sir James Sawyer, who, at last week's meeting, said he believed the scheme had been before the Hospital Committee for years. He knew that it was broached ten years ago, and it had been brought up from time to time ever since. But during the last few months the proposals had assumed definite shape. He hoped the scheme would be carried forward at once to a practical issue, for he could not doubt that it would prove successful. The Training Institution for Nurses in the Crescent had done a great deal of good, but since it had been established Birmingham had grown, and there was plenty of room for another institution of the kind, working side by side in friendly harmony with it. If there was any mistake at all in the establishment of the Training Institution, it was that it was not established under the shadow of a great hospital like the Queen's, which had led the way in many matters of hospital management and reform. They had taught their neighbours that special officers were an advantage; they were sneered at and imitated; and he thought it very likely that the General Hospital before long would be imitating them in the establishment of a nursing department. The Queen's Hospital was now a great deal more than a mere charity for the relief of the suffering poor: it was a school for the instruction of medical students; and its work would not be complete till it trained nurses as well as medical men. He hoped the scheme would be put into operation as soon as possible, and would regard it as a privilege to take part in the guarantee against any possible loss that was likely to arise.

### The Lords' Committee.

Since the Easter recess and Lord Sandhurst's attack of influenza the Lords' Committee has dragged somewhat. On one occasion none of the witnesses expected were present, and the Committee had to adjourn till another day. At the first meeting, Dr. Bridges, Medical Superintendent of the metropolitan district for poor-law relief, was examined at some length, and stated that there were in London twenty-four infirmaries, having 12,445 beds. There were also 4,000 beds in the workhouses. He did not think that was sufficient for the population. He urged that it would be a very desirable reform if all matrons of infirmaries were trained nurses, for while now there were trained nurses at the infirmaries, there were a number of matrons who were not qualified nurses. Although there was no friction, the influence of the matrons over the nurses was not sufficient from a professional point of view. He also thought that the infirmaries could be utilised with great advantage by the medical profession for clinical instruction. Mr. Charles Gross, Medical Superintendent at St. Saviour's Infirmary, East Dulwich, said that that was the largest in London, and had 786 beds. He also was in favour of the matrons being in all cases trained nurses.—Lord Thring: The effect of the infirmaries is greatly to tend to pauperise the people, is it not? Mr. Gross: Certainly.—Lord Thring: Yours is most startling evidence.—Mr. F. Horne, Secretary to the London Throat Hospital, Great Portland Street, and Mr. W. Abram, Secretary to the Central London Ophthalmic Hospital, Gray's Inn Road, were examined.

Dr. Knox, Medical Officer of Bethnal Green Workhouse, which was built about fifty years ago, said that the hospital contained 495 beds, the tenants of which were attended to by a day staff consisting of one Superintendent, eleven nurses, two male and two female imbecile attendants, and three infirm ward nurses. The night staff was composed of eight ordinary infirmary nurses, one male and one female imbecile attendant, and one female infirm ward nurse. The Matron had not received any hospital training, and only one of the nurses held any hospital certificate. He contended that the system of pauper help was a crying evil. They had as many as eighty paupers employed during the winter. He computed that each nurse had about four wards to look after, and in some cases as many as six on different floors. The average number of beds to each ward was nine or ten. Lord Cathcart: I suppose you enjoy at Bethnal Green the repute of being the very worst establishment of this kind in the metropolis?—Yes, I believe so.

Miss Louisa Twining gave some very interesting evidence in favour of trained nurses for workhouse infirmaries, and her opinion was listened to with obvious respect.

Dr. Thomas Savill, Medical Superintendent of Paddington Infirmary, said that the administration, discipline, and management of the infirmaries generally were very good, but there were two exceptions. The matrons were not always hospital-trained nurses. Although the Matron in Paddington Infirmary was hospital-trained, perhaps not twelve out of the twenty-four matrons had any such training. The medical staff was insufficient. Comparing the infirmary with St. Mary's Hospital, which was almost of the same size, he said the medical staff consisted of nine resident and nine visitors, whereas in the infirmary there were only three residents. He had not alluded to the number of students who help in the surgical cases. The hospital had many surgical cases but few nervous cases, while the reverse was the case in the infirmaries. It had been suggested that the infirmaries should co-operate with the hospitals and that the staff of the hospital might assist by coming and seeing the cases at the infirmaries. That kind of help was not what was required. The medical staff of the hospital had enough work to do already, and there were a great many men disconnected with any institution who ought to be considered. They required more residents who would do the drudgery of taking notes of the cases. The patients were all ill, bedridden, and not of the same type at all as those met with at the out-patient department of a general hospital—cases of rheumatism, bronchitis, and all stages of nervous diseases; and students might with very great advantage give their services in return for the advantage they would derive from these institutions. It had been argued that this would lead to increase in the expenditure, and that the patients did not like it, but at Paddington the experiment had been tried. It was the only infirmary which had tried the experiment of teaching from the cases, and the lessons learned from the experiment were that the patient did not mind the examination by the students, that it increased the interest of the medical staff, and that the cases were properly investigated.

The Committee then adjourned.

### Asylum Articles.

### I.—THE BUILDING.

The carriage turned in at an open gate. "This is Berrywood," said my companion.

"What! with open gates and no porter visible?"

"Yes, the open door system prevails largely here; you will find all the doors unlocked during the day, and there is not a single barred window throughout the building."

We were driving through a pretty wood, carpeted with bluebells and with a delicate undergrowth of luxuriant wild parsley. The nightingales were singing loudly, and through the trees one got glimpses of long stretches of woody country, and in the valley below could be seen the chimneys and smoke of Northampton.

"There is no wall round the grounds?" I said wonderingly, having in my mind recollections of other county asylums buried behind forbidding brick walls bristling with spikes.

"No," was the laughing reply; "only an iron railing a boy could climb over or a baby creep through. Yet we have never lost a patient or had a case of suicide since the system was started. See, there are the doctors and the attendants and the patients all playing cricket together."

This was a sufficiently strange introduction to an asylum visit, but wonder and admiration grew with the days. The asylum held 465 patients on the female side, and the building was divided into six chief wards. Each ward had its dining-room, dormitories, and lavatories complete. No. 1 was known as the acute ward, and here were the patients who had broken out, or, in the language of the medical officer, were "not very well to-day." The crockery was unbreakable, the ornaments few, in this ward; but in each succeeding ward there were more and more ornaments, and easy chairs, and birds, and pianos, till the home-look was quite acquired. As the patients improved in health, they were moved on to brighter surroundings, till finally they were landed in the "Home," a separate building in the wood where restraint was reduced to a minimum, and the inmates were regarded as convalescent and almost fit to be discharged. Of course, each patient did not kindly pass on to recovery in this orthodox way; there were cases which never got out of No. 1 ward; there were other cases which got out, but had to be put back again at intervals; but the system was thus to pass the patients on, and give them better surroundings as they grew able to appreciate them. The laundry for the whole asylum was on the female side, and the patients manned it completely save for two laundry-maids. All the clothes worn were made by the patients and nurses, and as many of the patients were destructive the garments had to be made not in dozens but in hundreds; they were stacked away in neat piles on shelves. New beds with wooden spring mattresses, like Howe's beds, were being gradually got in to supply the place of the old matting beds. Huge baths in rows were provided for each ward. The most scrupulous cleanliness prevailed everywhere, and the aspect of the whole building was cheerful and home-like compared with other institutions.

(*To be continued.*)

### Nursing Medals and Certificates.

ST. JOHN THE DIVINE.

THE medal pictured above is made of wood, and is worn by all the nurses working for St. John's community, slung on a blue ribbon hung round the neck. In 1883, owing to a disagreement at Charing Cross Hospital between the doctors and the St. John's community, a certain portion of the Sisters and nurses, headed by Miss Lloyd, seceded from the parent stem, and started a home at Drayton Gardens, and became known as the Sisters of St. John the Divine. The old community remain known as St. John the Evangelist; the uniform of the "Evangelist" nurses is brown; that of the "Divine" nurses is black; hence an irreverent public has come to distinguish between the two as "Black Johns" and "Brown Johns."

The Sisters at Drayton Gardens prospered steadily; they have now eight different branches of work, including a large hospital at Lewisham. Each probationer who passes successfully her first year in their midst is given the above cross to wear, but the cross has to be given up by any nurse leaving the service of the community. The Sisters' crosses are altogether different to those worn by the nurses. There are at present over 100 nurses who wear this cross, but their names are of course ever changing, and therefore cannot be given here.

### Appointments.

LEEDS HOSPITAL FOR WOMEN.—Miss Edith Sutcliffe, of Soho, has been elected Matron of this hospital.

NORTHERN COUNTIES HOSPITAL FOR INCURABLES.—Miss Jane P. Galloway, who trained at Edinburgh, has been elected Matron of this hospital.

MOUNTAIN SIDE HOSPITAL.—Miss Bessie Mushet, who trained at Dundee, and subsequently worked for the Blytheswood Institute, has been appointed Matron of this hospital at Montclair, New Jersey. Miss Mushet went to the States eight months ago with a patient, and, finding the New World congenial, applied for the above post and won it, though many American nurses competed.

STEPNEY INFIRMARY.—Miss Stockwell, who trained at St. Thomas's, and has since worked at Sussex County Hospital, has been appointed head nurse of the Stepney Infirmary. We congratulate the Board on their choice, and hope they will stedfastly pursue the path of reformation on which they have entered. The late nurse did excellent work by calling the attention of the guardians to glaring evils, and we hope her fate will not deter Nurse Stockwell from exposing the minor evils which still remain.

THE OTHER SIDE.

ONE of the worst things about a long illness is that it tends to make us think too much about self, about *my* pain, *my* weakness, *my* troubles, and so on. Now, this is a very bad thing for us, as we shall very likely all admit; for pain actually seems to get worse if we are for ever brooding over it; besides, so much thinking about ourselves makes us narrow-minded, and ever narrow-hearted—not at all the sort of persons that Christ, our Master, would like us to be. "This is all very true," you may say, "but how are we to help it?" Well, something in this way, I think. There is nothing but what has two sides, at the very least—the side which affects us, and the side which does not. The side which we see first is that which affects us—that is only natural; but we must take care that we do not see that alone,—nay, we must try and forget it altogether, and look out for the other side. Let me give one or two instances of what I mean.

There is a great noise in the street below, cabs and carriages rattling past, street-sellers crying their wares, children shouting and laughing. "Oh, my poor nerves! How it all worries me!" you cry. Then another thought comes. You try to think of the people themselves, instead of the effect their noise has on you; you try to fancy where they are all going, what they look like, how the breezes are blowing fresh and cool upon the outside 'bus passengers, as they go home tired from their work, how the children are "playing horses," or spinning their tops along the pavement,—until, with these pictures in your mind, you forget all about yourself and your poor nerves.

Or, again, you are wakened in the early morning by a thrush, who shouts out his insistent little "So it is," and "Bring it here," until you feel that you would like to throw things at him. "How can I sleep?" you exclaim angrily. Then suddenly you remember that *I* must not be talked of; and forthwith you begin to try to picture to yourself the little songster, with his speckled waistcoat, perched up in the tree, and somewhere near his patient mate, brooding over her nest, and her five blue treasures within. Is he singing to cheer her heart? Somehow the song sounds quite different, when you think of all this; it is no longer aggravating *me*, but charming *her*. Summer is coming again—"So it is! So it is! So it is!" he seems to say.

Try this simple little receipt, and I feel confident you will find—not only that the pain and weariness become less, but that your heart is growing larger and warmer—ay, and happier, day by day.

### Notes from Australia.

*(From our Own Correspondent.)*

Melbourne, May 5th.

The Charity Commission has been hard at work again. The question of nursing at the Homœopathic Hospital was introduced by a memorandum from the Under-Treasurer, forwarding copies of evidence and correspondence for the consideration of the Commission. The point had been originally raised by a report from Captain Evans, Inspector of Charities, who questioned the practice of allowing the nurses attached to the institution to be engaged by private patients as detrimental to the interests of the hospital patients. On the motion of Professor Morris, it was decided to reserve the reply until the final report of the Commission was being prepared. It is barely a twelvemonth since the Commission commenced its labours, and from that time the two months recess usual in Government departments has to be deducted, yet they have held 57 meetings, have examined 132 witnesses, have visited many up-country institutions, and will in one day complete their examination of the institutions in Melbourne and suburbs. The chief fault the Commissioners have found in Melbourne and elsewhere is that so many of the institutions have not proper fire escapes and engines; remembering in England how shop-assistants, girl-graduates, and asylum attendants are trained in their fire-drill, one recognises how remiss we are on this point over here. The Commissioners have visited the Magdalen Asylum, managed by Rev. Mother Kennedy, Sister O'Shea, and other Sisters. The asylum contained about 700 female inmates, of whom more than 300 had been deserted by their husbands, or were of drunken or dissolute habits. It also contained industrial school children to the number of 50, besides many children who had been deserted by their parents. There was in addition a day school composed of children whose parents reside in the locality. The greater portion of the funds necessary for the maintenance of the institution was derived from the labour of the inmates themselves, and out of a total expenditure of £10,178 last year, no less than £6,796 was obtained from the laundry work done in the institution. No Protestant community in the colony can show anything like such a record of good work. The Sisters have obtained the washing from the different lines of steamers. Their cart is on the pier waiting arrival, and then all speed is made to get an enormous wash done in about thirty-six hours. Their method and good organization is the admiration of all who know about their work.

There has been an outbreak of typhoid at Kew Lunatic Asylum, and no wonder, it is so shamefully overcrowded. It was built to hold 700 patients, and contains 1,300. Five medical officers have to be appointed at Melbourne Hospital, and it is done by subscribers' votes, an absurd way, the evil of which is being discussed in the Press. By next year several lady medical students will have graduated here and be eligible for election, unless special rules are made to exclude them. The finances of the Melbourne Hospital are in an unsatisfactory state. At the meeting of the Committee of that institution yesterday, Mr. Webster, in submitting the report of the Finance Committee, remarked that the subject required the most serious consideration. He was afraid that at the end of six months there would be a debit balance of nearly £10,000. The funds were gradually going down, a disgraceful fact considering how the colony is going up.

There has been a squabble at the Austin Hospital; a patient complained of the head nurse, and the complaint was openly investigated in the ward in the presence of the other patients. The nurse was exonerated from all blame, but her feelings were hurt by the public manner in which the inquiry was made. Perhaps the best apology for all our shortcomings over here is found in the census report just issued. In 1881 the Melbourne population was 282,000; now, in 1891, it is 489,000. When a town grows at this rate, it is like a reedy youth—sure to be rather unhealthy, though, doubtless, with a strong and sturdy future before it.

The Little Sisters of the Poor have opened another wing to their Home at Northcote. The main portion of the home was opened last June, and afforded accommodation for 154 males and 51 females. With the opening of the new wing the Sisters expect to increase the number of patients to between 250 and 300, all of whom will be housed in the new building. It is only a few years ago that Sister Beatrix Marie came over and established the order here, now there are eighteen sisters who do the whole work of the establishment. The new building is handsome and complete in every detail. Every drain is protected by an elaborate series of catch traps, and the same precautions have been taken in the case of interior sinks. The lavatories and bathrooms are in separate wings, connected with the main building by arcades, so that complete isolation is secured without any discomfort to the occupants. There is also a very simple and yet perfect method of flushing drains every morning. The value of these precautions has been fully recognised by the Hon. Medical Officer to the order, Dr. O'Sullivan, who mentions that the recovery in certain cases of operation within the Home has been astonishingly rapid, patients reaching a stage in a fortnight which could hardly have been expected under at least five weeks in any other institution. The total cost of building and furnishing the Home up to date has been £28,700, of which £19,450 has been raised by public subscriptions, leaving a debit balance of of £9,250. As the Sisters receive no Government grant, and are not included in the Hospital Sunday collections, the Home is dependent purely upon the donations of the charitable public. It has this additional distinction, that not one penny of the public money is applied to any other purpose than the direct benefit of the aged and infirm poor. There are no servants. The whole of the menial work is done by the Sisters, who, in their noble vocation of mercy, do not disdain to scrub floors. Hardly a penny is spent on food, most of which is collected by the Sisters from various restaurants.

### Notes and Queries.

### Queries.

(18)—Where can I buy a district nurse's basket?—*Nurse G.*

### Answers.

(16).—There is a free home for consumptives. Address, St. Michael's Home, Axbridge, Somerset. It is managed by the Kilburn Sisters, and only members of the Church of England are admitted.

(16)—Mrs. Mary Lynch, Tangland Lodge, Tatsfield, Westerham, would take charge (free) of a consumptive patient for a week or more.

(17)—There is a one-horse ambulance at St. Bartholomew's Hospital. The St. John Ambulance, Clerkenwell, E.C., keep carriages for moving the sick.

*Quero.*—The Jenny Lind Infirmary, Norwich, but you may have to wait for a vacancy. You might try the Ipswich Hospital.

*B. M.*—News inserted this week; we will be glad to have the article you propose and will pay for it at the usual rate.

*G. M. E.*—We must refer you to our back numbers, in which are many articles on nursing in New York and Canada. We chronicle this week an appointment gained by a Scotch nurse who emigrated a few months ago. We would specially refer you to "A Letter from Montreal," in our issue for September 20th, 1890, and "A Letter from Kansas," May 9th, 1891.

*J. O.*—We received so many letters on the subject we were unable to print yours, though we agreed with it.

*H. B.*—The Edinburgh Royal Maternity Hospital. We believe the fee is five guineas for three months, and 8s. a week for board and lodging.

*Letters Wanted.*—Will the nurse who sent in a notice under this heading state to what institution or association she belongs, and her "want" will then be inserted.

*Asylum Nurse.*—Apply to the Secretary, Medico-Psychological Association, Hanwell Asylum, London, W.

*A Novice in Private Nursing.*—Certainly you should make the gruel yourself; any duties connected with the well-being of your patient fall on you. The engagement is for the lunar month as a rule.

*Nurse Lucy.*—See answer to G. M. E. We hope to publish a letter from New York next week.

*C. P.*—Your query is an advertisement in disguise.

### Everybody's Opinion.

*[Correspondence on all subjects is invited, but we cannot in any way be responsible for the opinions expressed by our correspondents. No communications can be entertained if the name and address of the correspondent is not given, or unless one side of the paper only be written on.]*

### THE DULNESS OF DOMESTIC SERVICE.

"A Reader of The Hospital" writes: I see in last week's Hospital a "Mistress of Many Years' Standing" complaining of the outing domestic servants want, and I think from the tone of her letter she would never let her domestics out one half as much as she says domestics ask for. In fact, I rather doubt whether she would let them have the breath of life. It is not an English servant she ought to have; it is a slave she wants, I should think. A pity she cannot have a servant that she could wind up like an eight-day clock, so that she could work night and day. I feel quite sure she would never let her run down for the want of winding. As regards servants not working between meals, I do not know where servants will find such situations, for I have been in five situations, and three out of the five I scarcely had time to eat my food. These three I chanced to get in succession, after being five years in my situation previous, but I did not stay long in any of the three—a month in the two first and six months in the third—so perhaps the "Mistress of Many Years' Standing" gives her domestic servants too much work to do, and then she fancies they are dull and slow.

### BOSTON INFIRMARY.

"Ohna" writes: We are all sorry to learn that the Union Infirmary at Boston is no longer served by a trained nurse. It is a grievous pity that this retrograde step should have been made in the interests of one of the aldermen of Boston. It is specially serious on account of the midwifery department of this Infirmary.

### DISTRICT NURSING.

"E. H." writes: I shall feel glad if you will allow me a little space in "The Hospital" to draw attention to what I consider is "riding a good horse to death." District nursing is an excellent thing in its way, and when properly carried out is a great boon to the sick poor who are unable to pay for skilled nursing, but it seems to me from experience that the thing is carried too far now, and that some ladies who are quite unfitted for the task employ a district nurse, constitute themselves lady superintendents, and actually do more harm than good by making the nurse work under her rather than under the doctor's directions. Some do it for self-glory, others from a mistaken idea of "doing good," whilst I knew of one weathly "*parvenue*," who considered it would be a stepping-stone to good society. Her knowledge of nursing was of a most scanty order, though not so in her own estimation, as she one day said to the nurse, "You look down on me, but nursing in my own family, common sense, and observation have done for me what training has done for you." How much good it would have done this most learned "Superintendent" to be a probationer: she would perhaps then have found that all she knew would go into a very tiny nutshell. Doctors feared to employ this district nurse, as they knew the "Lady Super" would not always allow their directions to be carried out. Surely it must be very galling to any trained nurse to work under such a woman, and I cannot imagine any nurse "who is worth her salt" doing it. Truly this growing evil could easily be put down if nurses would refuse to work under any one but medical men or trained Superintendents. How can a woman who has had no training direct one who has? Might just as well get a person who has never heard or read a word of French, to teach a girl who has lived eight years in France. Truly a little knowledge is a dangerous thing. Let district nursing be carried out properly in a right spirit, it is, indeed, a noble undertaking; but nothing can be much more harmful to the profession, the people, and the place, than the same thing badly done, viz., from individual, vain, selfish motives.

### Presentations.

The committee of working men and their wives who help support the Hull Royal Infirmary lately visited the institution, and were shown over by the Matron. They were so pleased with all they saw that at a meeting of the Executive Committee, held at the Infirmary on Saturday, the Chairman (Mr. W. Fawcett) presented to the Children's Ward, on behalf of the wives of the members, a beautiful mahogany swing cradle, as a memento of their visit to the institution. The members of the Committee also expressed their gratification by presenting to the Lady Superintendent (Miss Cox) a Russian leather satchel, to the housekeeper (Miss Phillips) a silver pen and pencil case, and to the collector (Mr. Thackeray) a silver pencil case, as mementoes of the visit, and a slight recognition of their kind and courteous attention. A hearty vote of thanks was unanimously accorded to the Board of Management for the opportunity afforded, and the excellent arrangements made for such a large number of visitors.

### Montrose Asylum.

The following details from the report just issued of this Asylum may be of interest to such of our readers as are attendants: Dr. Howden stated that the number of patients on the register on May 15th was 542. Sixty-seven patients were discharged, 48 of whom had recovered, while 17 were relieved, and two not improved. Twenty-four died. Of those admitted, 99 were new cases, while 22 were re-admissions. Twenty-seven were private patients, and 94 rates supported. Eighteen were in apparently good bodily health on admission, 57 in indifferent and 46 in positively bad health. Fifty-nine were at the time of admission considered to be possibly capable of recovering mentally, though many of them laboured under incurable bodily disease; on the other hand, in 62 cases no hope could be entertained of recovery from their mental disorder. There were no suicides during the year, and the only injuries recorded were, one case of fractured ribs, one a fracture of a toe, one a fracture of the clavicle, and one fracture of the neck of the femur. A female patient was confined to her room on three occasions, and for a total period of four hours, during paroxysms of maniacal excitement. Restraint with the straight jacket was adopted in two instances to prevent self-mutilation, in two to prevent the removal of surgical dressing, and in one case for persistently tearing his bed-clothes with his hands and his teeth. In the latter case the patient was quite pleased with his new dress, and was most unwilling to part with it at the end of five days, when his destructive tendencies had in a great measure died out. In November we lost a valued officer by the death of Miss Burness, the Matron, who had been in the service of the Institution for the long period of 34 years. Her place has been filled by the appointment of Miss Chappell, who, in addition to other strong recommendations, brought with her extensive experience in sick-nursing gained in the Royal Edinburgh Infirmary. The changes amongst the attendants have been exceptionally numerous during the year. Three female nurses left, and twenty male attendants. Two of the nurses left to be married, and the other to go home. Of the men, nine left for other situations, two on account of bad health, five were dismissed for returning after leave of absence the worse for drink, one for striking a patient, and one for disobedience of orders and general unfitness, and two for incapacity.

### The Message of the Roses.

"Such a hamper of roses!"

The news went from Sister to nurse, from nurse to probationer; and presently everybody in the ward knew all about it, for the roses themselves told the story as soon as the hamper was unpacked. There were stacks of them, a lavish wealth of regal blossoms. And not shabby, stunted specimens which by any other name would most distinctly have not smelled half so sweet, but large, generously cultivated, glorious roses. Some deep, deep red, well nigh black in their ruddy gloom; others, heavy, rich, creamy yellow; and yet others, so snowy in their whiteness, so pure, as if formed to wreathe the brows of angels or the meek dead.

"Whoever sent these roses did not grudge their very best for the sick!" murmured a Sister as she gathered up in her hands a great bunch, and moved away down the ward, and out of it, telling herself that somebody she knew would rejoice over her prizes—somebody who lay wan and spent in the paying patients' room, and why not roses for her as well as for the sick poor?

"Look up, my dear!" At the kindly Sister's softly modulated voice, a pair of dark-grey, pathetic eyes opened wide; eyes that were homes of trouble and unrest, but into which a great flash of welcoming joy stole at sight of the mass of colour and perfume that lay upon the quilt, close up to their owner's lips.

"Oh!" There was a glad cry of ecstasy, and then the beautiful roses were gathered still closer, and kissed as if they were living things. "Roses from home! So they sent them, did they?" and the patient looked inquiringly up into the Sister's calm face.

"I don't know where they came from. I did not hear; but they are truly superb!"

"They came from my old home, I tell you," feebly persisted the patient. "Do you suppose they are strangers to me? I recognise each one as an old friend. See!" and lifting each rose caressingly, she repeated to the Sister the name of its variety. "And father grew them, every one of them," she ended simply.

The Sister gave the speaker a curious glance. Was the girl wandering in her mind? she silently thought. But the dark grey eyes were tranquil enough, and the Sister left the room, returning in a few minutes to hold the card enclosed in the hamper before the patient's face.

"I told you so! They are Granby roses!" Then the little story was poured out hysterically. The music teacher, overworked and under-fed, from the cheap suburban school, who lay prostrated by many ills, was the runaway daughter of a wealthy luxurious home—its only child, rendered frantic, with passionate indignation, at the unexpected advent of a stepmother.

"And I'm sorry, now, that I did run away!" ended the weak girl with a remorseful wail. "The Granby roses have voices which cry out for me."

"And what is their message, my child?" asked the Sister, laying her cool hand on the girl's flushed brow.

"They are calling, 'Come home! Come home!' Ah, if I *could*; but it is too late."

"Not so! Already you are creeping out of the dark valley. God is going to give you another chance. Will you again refuse to bear that special cross He designed for you—that cross you threw from you rebelling at its weight?"

Before the Sister's grave, questioning eyes those of the patient fell, and her thin fingers worked nervously with the fragrant petals of one—a very queen—of the heap of roses.

"I should *try*, but ——" she whispered feebly, then ceased abruptly.

It was enough. Before the summer's roses had faded the paying patient's room was vacant; and the Sister's heart was rejoicing over the good work she had, on her own responsibility, achieved. The petted daughter was again at home; its idol as of old. The taste of life's hardships had been a bitter one; but tonics are wholesome, and necessary as well. The spoilt girl was braced mentally, and—for practical knowledge is a splendid teacher—she knew, through all the future, that the other side of life from her own prosperous path was full of gloom that wealth was meant to lighten.

And that special cross of her's? It had disappeared. The stepmother proved to be a meek, gentle soul, with a perfect genius for mothering a delicate, highly-strung girl, and the natures of the two women fitted in to a curious nicety.

"How I shall always love and bless the roses!" cried the happy girl.

"How I shall always love and bless the good hospital Sister who helped you home, dear!" was the echo—with a difference—tenderly spoken by the now loved stepmother.

### To the Fore.

When the weak ones help are needing,
When the strife is waxing sore,
Not in vain shall be their pleading,
Maids of England, to the fore.

When the weary ones are sinking,
Feeling they can fight no more,
Ne'er of self or glory thinking,
Maids of England, to the fore.

When the sick ones and the dying,
Touchingly our aid implore,
Not in vain shall be their crying,
Maids of England, to the fore.

Hear the voices of your brothers,
Calling to you evermore;
When there's work to do for others,
Maids of England, to the fore.

England's women, greater glory
Than your fathers gained of yore
Shall be yours in heavenly story:
Maids of England, to the fore!

V. M. Walsh.

### Amusements and Relaxation.

SPECIAL NOTICE TO CORRESPONDENTS.

**Second Quarterly Word Competition commenced April 4th, ends June 27th, 1891.**

Competitors can enter for *all* quarterly competitions, but no competitor can take more than *one first prize* or *two* prizes of any kind during the year.

The words for dissection for this, the THIRTEENTH week of the quarter, being

"ARGENTINE."

| Names. | June 17th. | Totals. | Names. | June 17th. | Totals. |
|---|---|---|---|---|---|
| Christie | 21 | 401 | Ivanhoe | 16 | 342 |
| Patience | — | 214 | Weta | — | 147 |
| Agamemnon | — | 367 | Lady Betty | — | — |
| Hope | 22 | 407 | Mortal | — | 76 |
| Reldas | 22 | 40/ | Little Eliza | — | 147 |
| Lightowlers | — | — | Dove | — | 95 |
| Nurse J. S. | 16 | 335 | Ladybird | — | 141 |
| Qu'appelle | — | 170 | Psyche | 18 | 352 |
| Jenny Wren | 15 | 325 | Ugug | — | 229 |
| Wyameris | 21 | 405 | Harrie | — | — |
| Paignton | 19 | 341 | Grannie | 17 | 324 |
| Theta | 17 | 223 | Eale | — | 169 |
| Success | — | 17 | Grimalkin | — | 53 |
| Tired | — | 136 | Nurse G. P. | 11 | 120 |
| M. G. | — | 188 | | | |

**Notices to Correspondents.**

**N.B.—Third Quarterly Word Competition commences July 4th, 1891.**

All letters referring to this page which do not arrive at 140, Strand, London, W.C., by the *first post on Thursdays*, and are not addressed PRIZE EDITOR, will in future be disqualified and disregarded.

## Figures and Tables

**Figure f1:**
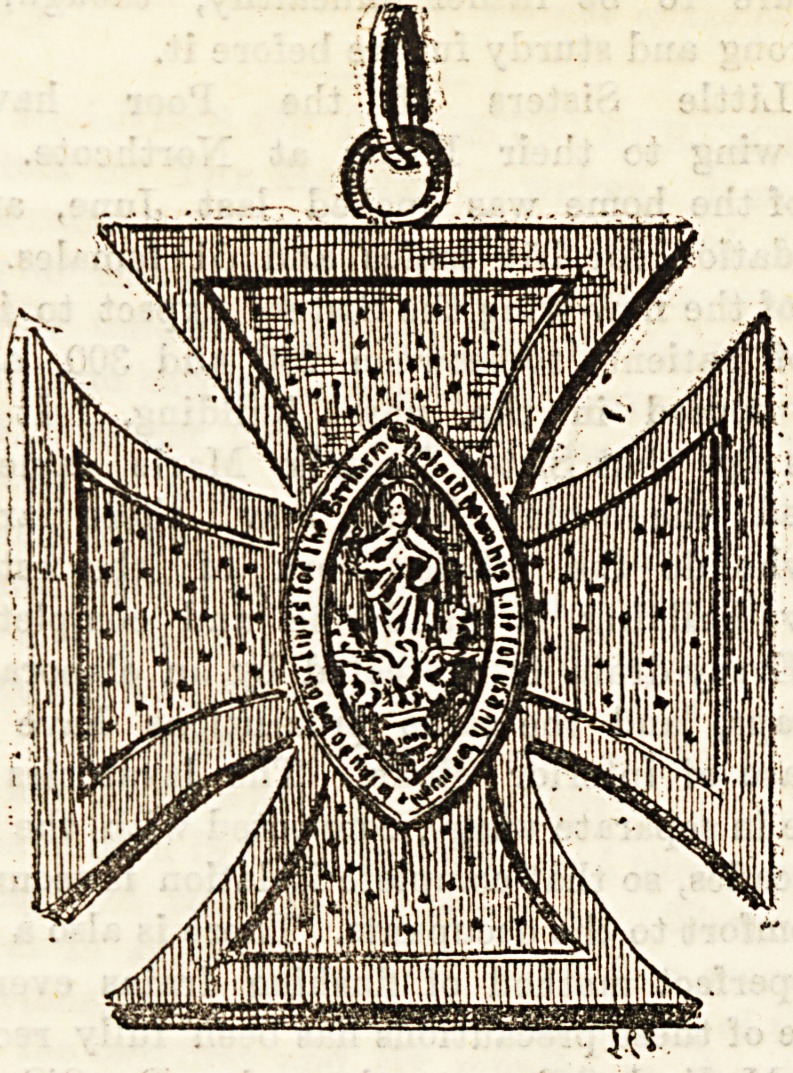


**Figure f2:**